# Availability of and Ease of Access to Calorie Information on Restaurant Websites

**DOI:** 10.1371/journal.pone.0072009

**Published:** 2013-08-20

**Authors:** Gary G. Bennett, Dori M. Steinberg, Michele G. Lanpher, Sandy Askew, Ilana B. Lane, Erica L. Levine, Melody S. Goodman, Perry B. Foley

**Affiliations:** 1 Duke Obesity Prevention Program, Duke Global Health Institute, Duke University, Durham, North Carolina, United States of America; 2 Department of Psychology and Neuroscience, Duke University, Durham, North Carolina, United States of America; 3 Department of Surgery, Division of Public Health Sciences, Washington University in St. Louis School of Medicine, St. Louis, Missouri, United States of America; University of Missouri-Kansas City, United States of America

## Abstract

**Objective:**

Offering calories on restaurant websites might be particularly important for consumer meal planning, but the availability of and ease of accessing this information are unknown.

**Methods:**

We assessed websites for the top 100 U.S. chain restaurants to determine the availability of and ease of access to calorie information as well as website design characteristics. We also examined potential predictors of calorie availability and ease of access.

**Results:**

Eighty-two percent of restaurants provided calorie information on their websites; 25% presented calories on a mobile-formatted website. On average, calories could be accessed in 2.35±0.99 clicks. About half of sites (51.2%) linked to calorie information via the homepage. Fewer than half had a separate section identifying healthful options (46.3%), or utilized interactive meal planning tools (35.4%). Quick service/fast casual, larger restaurants, and those with less expensive entrées and lower revenue were more likely to make calorie information available. There were no predictors of ease of access.

**Conclusion:**

Calorie information is both available and largely accessible on the websites of America’s leading restaurants. It is unclear whether consumer behavior is affected by the variability in the presentation of calorie information.

## Introduction

The average American diet is increasingly comprised of meals consumed away from home. [1] These meals are potent drivers of the U.S. obesity epidemic – a single restaurant meal each week can add two pounds annually to the average American’s waistline. [2] To assist consumers in making more healthful, lower calorie purchasing decisions, restaurants are increasingly being encouraged to offer calorie data on their in-store menus. [3] While many large restaurant chains currently provide nutritional information on request, pending federal regulations will require restaurants with more than 20 locations to label their on-site menus with calorie information. [4] Several large chains, including Panera Bread and McDonald’s, have proactively announced plans to label their in-store menus with calorie data prior to the federal mandate.

Restaurants are also increasingly offering calorie and nutrition information on their websites.[5]–[7] This is important as recent reports indicate that 81% of adults in the U.S. have Internet access and 59% of adults use the Internet to search for health information. [8] Regarding weight control topics specifically, Blacks and Latinos as well as those with higher levels of education are more likely to search for weight control information online, compared to Whites and those with less education. [8] Access to website-based calorie information might be particularly important for groups who are disproportionately affected by obesity. [9] This information can be used for planning purposes, allowing consumers to identify restaurants and meals with lower calorie options prior to the point of purchase.

Despite the increase in availability of calorie information, its ease of access on restaurant websites remains undetermined. Restaurant websites vary considerably in visual complexity, the range of information provided, navigation, usability, and adherence to best practice design principles. It is unclear whether the design of restaurant websites inhibits the ease of accessing calorie information. Thus, the purpose of this study was to assess both the availability of and ease of access to calorie information on the websites of the top 100 U.S. restaurants (2010). Given the rapid shift towards using mobile devices for Internet access, [10] the availability of calorie information on mobile-formatted versions of restaurant websites was also assessed.

## Methods

Restaurants were identified in the Technomic, Inc. 2011 report of the top 100 U.S. chain restaurants, by revenue. [11] Data were collected in January 2012 by eight reviewers who were each randomly assigned to review 25 restaurants; consequently, each restaurant was reviewed twice. We used multiple web browsers on laptop computers to examine restaurant websites. A separate reviewer evaluated the availability of calories on a mobile-formatted website. Any discrepancies were resolved through consensus.

Raters examined the availability of and ease of access to calorie information. Calorie information was considered available if the website included menu items that were presented with associated calorie data. For sites that provided calorie data we also assessed additional nutritional information, including carbohydrates, protein, fiber, sodium, saturated fat, total fat, sugar, cholesterol, and calcium. Ease of access was operationalized as the minimum number of clicks necessary from the website homepage to view calorie information for any food item. For sites that offered calorie information through multiple navigational paths, all available paths were assessed and the minimum number of necessary clicks was recorded. Further assessment regarding website characteristics included the location and labeling of homepage links to calorie information, whether websites included a link labeled “nutrition” or “calories” in their primary navigation menus, and/or whether calorie information was available in downloadable documents. In addition, we assessed whether restaurant websites featured healthy food items. Restaurants collated these sections in a variety of ways (e.g., foods low in calories or sodium, foods high in protein) and identified them using unique graphics and/or text. Reviewers also rated the presence of interactive tools (e.g., “build-a-meal”), which allow consumers to select various food options and receive an automatic calculation of the total calorie content. Finally, among those restaurants that provided web-based calorie information, availability of a mobile version was assessed via multiple mobile operating systems to examine whether restaurants also presented calorie information on mobile-formatted websites. We did not assess ease of accessing calorie data on mobile-formatted websites.

We also examined several restaurant-level predictors of availability and ease of access, including chain revenue, segment, and number of units in 2012. To assess segment, we categorized restaurants based on Technomic’s industry classification [11]: quick service (e.g., McDonalds), fast casual (e.g., Panera Bread), and full service, which included casual dining (e.g., Olive Garden) and fine dining (e.g., Ruth’s Chris Steak House). We assessed average entrée cost using price classifications from Yelp.com (≥$11.00 vs. $10.00 or less) and categorized cuisine type using Technomic’s structure [11]: asian, bakery café, beverage, cafeteria/buffet, chicken, donut, hamburger, family style, frozen desserts, italian, mexican, other sandwich, pizza, seafood, snack, steak, and varied menu.

### Statistical Analyses

We first used descriptive statistics to depict the extent of calorie availability and ease of access. We estimated simple logistic and linear regression models to examine associations between each of the restaurant characteristics and the availability of and ease of access to calorie information. Availability was dummy coded to *available* or *not available* and ease of access was treated as a continuous variable (number of clicks). Characteristics found to be statistically significant in the simple logistic/linear regressions were entered into a multiple logistic/linear regression model, and non-significant variables were removed one at a time based on their significance level (p>.10). All analyses were conducted using SAS Version 9.3 (Cary, NC) and SPSS Version 20 (Chicago, IL).

## Results

Of the 100 restaurants listed, 46 were quick service, 13 were fast casual, and 41 were full service (which included 40 casual dining and one fine dining chain). Most restaurants (49%) served one of the following cuisine types: hamburger (15), family style (11), pizza (10), or varied menu (14). All restaurants had websites; 82% of those presented calorie information. A higher percentage of quick service and fast casual restaurants provided calorie information (95.7% and 92.3% respectively) than did full service establishments (65.0%). Seventy-six percent presented calorie information for beverages. We found that if calorie data was present, a high percentage (range: 82–100%) of restaurants also provided additional data on macro- and micronutrients. However, calcium was less frequently available than the other nutrients (47% of websites). Only 25% of those restaurants with web-based calorie information had calorie information available on a mobile-formatted website.

On average, calorie information could be accessed in 2.35±0.99 clicks (median 2.00, IQR 2.00–3.00). About half of the sites reviewed (51.2%) had at least one link labeled “nutrition” or “calories” on the homepage. However, a smaller proportion (35.4%) presented this information on their primary navigational menu. Nearly half had a separate healthy eating section (46.3%). Forty percent of sites clearly identified healthy foods, and 35.4% had an interactive “build-a-meal” feature. Approximately two-thirds of sites (68.3%) made calorie information available as a downloadable document (e.g., PDF), and of those, 35.3% did not provide calorie information in any other format. Almost two-thirds of the restaurants (64.6%) had at least two of the five features reviewed: 1) information accessible as a downloadable document, 2) primary navigation link labeled nutrition or calories, 3) interactive meal planning tools, 4) separate healthy eating section, 5) healthy foods clearly identified in nutrition section. Only four sites had all five features available. Detailed information for each of the 100 restaurants reviewed can be found in Table S1. Restaurants are ordered by revenue (highest to lowest).

### Predictors of Availability and Ease of Access

Table 1 highlights the predictors of availability and ease of access from simple logistic regression models. There was a significant association between a chain’s number of units and its likelihood of providing calorie information, such that restaurants with greater units were less likely to provide calorie information than were restaurants with fewer units [OR (95% CI): 0.998 (0.996, 0.999); p = .02]. Entrée cost category was also a significant predictor of availability; on average, restaurants with more expensive entrées had lower odds of having calorie information available [OR (95% CI): 0.21 (0.07, 0.65); p = .01]. Quick service and fast casual restaurants were more likely to provide calorie information than were full service restaurants [OR (95% CI): 10.77 (2.87, 40.47); p<.001. Higher chain revenue was marginally associated with lower likelihood of availability of calorie information [OR (95% CI): 0.98 (0.96, 1.00); p = .06].

**Table 1:** Associations of restaurant characteristics with the availability of and ease of access to calorie information.

	Availability of calorie information		Ease of access to calorie information		Availability of calorie information	
	Unadjusted models (n = 100)		Unadjusted models (n = 82)		Adjusted models(n = 100)	
	OR [95% CI]	*p-*value	M (SE)	*p-*value	OR [95% CI]	*p-*value
Revenue	0.98 [0.96, 1.00]	0.06	−0.001 (0.004)	0.79	0.98 [0.96, 1.00]	0.05
Number of units	0.996 [0.998, 0.999]	0.02	0.000 (0.000)	0.92	–	
Entrée cost($11+ vs. $10 or less)	0.21 [0.07, 0.65]	0.01	0.093 (0.229)	0.69	–	
Restaurant type						
Quick service/fast casual^a^	10.77 [2.87, 40.47]	<.001	−0.045 (0.235)	0.85	11.84 [3.02, 46.45]	<.001
Full service^b^	reference		reference		reference	

a N (%) = 59 (59) for availability models; N (%) = 56 (68.3) for ease of access model.

b N (%) = 41 (41) for availability models; N (%) = 26 (31.7) for ease of access model.

In multivariate models examining availability of calorie information, only revenue and segment remained significant. Chains with higher revenues [OR (95% CI): 0.98 (0.96, 1.00); p = 0.05) were marginally less likely to make calorie information available online. Additionally, compared to full service restaurants, quick service/fast casual restaurants were significantly more likely to provide calorie information online [OR (95% CI): 11.84 (3.02, 46.45); p<.001). There were no statistically significant predictors of ease of access in the simple or multivariable linear regression models.

## Discussion

Calorie information is both available and largely accessible on the websites of America’s leading chain restaurants. Within about two clicks, calorie information was accessible on 82% of restaurant websites. However, calorie data are less available for smaller chains, for those with more expensive entrées, and among chains in particular segments. Full service restaurants were less likely to provide calorie information than were quick service and fast casual restaurants. This pattern may be the result of greater variability of food combinations and meal options at full service restaurants, at which customers sit and eat for longer durations, often ordering multiple courses. In contrast, at quick service or fast casual restaurants, food options may be more limited and the purchase of a single item more common. As a result, customers at full service restaurants may be more likely to consume a greater number of calories compared to the average customer at quick service or fast casual restaurants. Because of these differences, full service restaurants may be less likely to provide – either intentionally or unintentionally – calorie information on their websites. Notably, no statistically significant predictors of accessing calorie information were identified. Many Americans have limited knowledge about the calorie content of meals consumed away from home, making the widespread availability of and ease of access to web-based calorie information a potential boon for consumers.

The pending federal regulations mandating calorie labeling at the point of purchase are premised on the supposition that consumers will use this information to shift their purchases towards lower calorie, more healthful food and beverage items. Indeed, consumers appear to hold positive public perceptions about menu labeling, [12] and report greater awareness of calorie information when exposed to labeling. [13], [14] Interestingly, racial/ethnic minorities report greater support for calorie labeling in restaurants compared to Whites, [12] suggesting that the federal mandate could impact those most at risk for obesity. Emerging evidence on the effect of menu labeling on consumers’ decisions at the point of purchase are mixed. Despite some positive support indicating a reduction in average calories per transaction, [15], [16] the majority of evidence from real-world evaluation studies suggests that calorie labeling does not impact decisions towards lower calorie foods at the point of purchase.[17]–[19] One might hypothesize that exposure to calorie information at the point of purchase may come too late to shift purchasing decisions, particularly for those consumers who are trying to improve their dietary consumption by reducing calorie intake. In contrast, web-based access to calorie information may promote shifts in restaurant selection and/or food choices prior to the point of purchase, thus potentially having an important impact on consumers’ purchasing decisions.

Our findings demonstrate that online calorie information can be accessed reasonably quickly, but they do not indicate how easily calorie data can be used for planning purposes. It is striking that restaurant websites present calorie information using a wide array of interfaces that vary considerably in design and functionality. For example, many restaurant websites are designed to offer calorie data only about individual menu items. Some do this by only listing calorie values, while others present complete nutritional information (including macro- and micronutrients) – either using the traditional USDA food label format or proprietary designs. Only one-third of sites utilized interactive “build-a-meal” functionality, which allows consumers to pick several food options and receive an automatic computation of the total calorie count. Only about half of the included links were clearly labeled as “calories” or “nutrition;” only about a third of these clearly labeled links were placed in websites’ primary navigation menus, which facilitates greater usability. [20].

This wide variety in interface designs has potential to cause confusion among consumers (see Table S1). Consider the example of a consumer who is evaluating calorie information at five leading hamburger chains (Wendy’s, SONIC, Carl’s Jr., Red Robin, Five Guys). At the time of data collection, all provided calorie data on their websites, but only two included a clearly labeled link in their primary navigation, three included a build-a-meal feature, two offered a “healthy eating” section, and two presented mobile-formatted calorie information. In addition to promoting confusion, variability in interfaces might minimize consumers’ ability to compare restaurant websites for the purposes of evaluating potential food options. At present, there is no evidence regarding which designs and/or features on restaurant websites facilitate optimal consumer usability. More work is necessary to determine whether design features known to optimize website usability (e.g., placing important content on the homepage, minimizing clicks, ease of learning, efficiency of use, memorability) [20] result in greater uptake and recall of web-based calorie information.

An additional area of concern is that so few restaurants offered calorie information using a mobile phone-formatted website. While most smartphones can access all websites, usability is greatly optimized when sites are formatted specifically for mobile presentation. Smartphone access to calorie information might be particularly important for those at high obesity risk. Racial/ethnic minorities, for example, have the highest obesity rates and are disproportionately more likely than Whites to own and use smartphones, including for accessing information about health and weight loss. [8] Providing appropriately formatted calorie content for mobile devices allows the data to be easily used both before and at the point of purchase.

Several issues should impact interpretations drawn from these findings. This study provided only a snapshot of the availability of and ease of access to calorie information on restaurant websites, and examined only restaurants with the largest revenues. Restaurants frequently adapt their websites’ features and interfaces, which could change these results, so it is not possible to infer whether these findings would still hold if the data were collected at a different time. Finally, this study was not able to determine the extent of utilization on the sampled websites.

As patterns continue to shift towards more meals consumed outside the home, [21] there have been parallel increases in the number of consumers who go online to investigate restaurants, [22] and in the number of online services that assist consumers in choosing restaurant options. The online availability of and ease of access to calorie information can provide important restaurant and meal-planning tools, especially for consumers who are attempting to modify their diets. Web-based calorie information can facilitate informed decision-making and has the potential to shift consumer choices to lower calorie, more nutritious meal options. Future research should assess whether and how online information acts as a prompt to influence food purchases at the point of purchase. Similarly, it would be useful to better understand the impact of various site features of restaurant websites on customers’ food orders. This might ultimately assist us in designing strategies to positively influence food purchasing decisions.

## Supporting Information

Table S1 **Availability of website features by restaurant among the top 100 U.S. chain restaurants, by revenue.**
(DOC)
