# Supplementary material for: Availability of and Ease of Access to Calorie Information on Restaurant Websites
Source: PLoS One. 2013 Aug 20;8(8):e72009. doi: 10.1371/journal.pone.0072009 (PMC3747846; doi:10.1371/journal.pone.0072009)
Supplement: Table S1 — Availability of website features by restaurant among the top 100 U.S. chain restaurants, by revenue. (DOC) [file pone.0072009.s001.doc]

|  | **Table S1. Availability of website features by restaurant among the top 100 U.S. chain restaurants, by revenue** | | | | | | | |  |
| --- | --- | --- | --- | --- | --- | --- | --- | --- | --- |
|  | **Restaurant** | **Calorie Information Available** | **Information Accessible as a PDF^1^** | **Primary Navigation link labeled "nutrition" or "calories"^1^** | **Interactive meal-planning tools available^1^** | **Separate healthy eating section available^2^** | **Healthy foods clearly identified in nutrition section^1^** | **Calorie information available on mobile device^3^** | **Segment type^4^** |
| 1 | McDonald's | X | X |  | X | X |  | X | QS |
| 2 | Subway | X | X | X | X | X | X | X | QS |
| 3 | Starbucks | X |  | X |  | X | X | n/a | QS |
| 4 | Burger King | X | X | X | X |  | X | X | QS |
| 5 | Wendy's Old Fashioned Hamburgers | X | X |  | X | X |  | X | QS |
| 6 | Taco Bell | X |  | X | X |  | X | X | QS |
| 7 | Dunkin Donuts | X | X | X |  | X | X | n/a | QS |
| 8 | Pizza Hut | X |  |  |  |  |  |  | QS |
| 9 | KFC | X | X | X |  | X |  | n/a | QS |
| 10 | Applebee's Neighborhood Grill & Bar | X | X |  |  | X | X | n/a | FS |
| 11 | SONIC Drive-ins | X | X | X |  | X |  |  | QS |
| 12 | Chili's Grill & Bar | X | X |  |  | X |  |  | FS |
| 13 | Chick-fil-A | X |  |  | X | X |  | X | QS |
| 14 | Olive Garden | X | X |  |  | X | X | X | FS |
| 15 | Domino's Pizza | X | X |  | X | X |  |  | QS |
| 16 | Arby's | X | X |  | X | X |  | X | QS |
| 17 | Jack in the Box | X | X | X | X | X | X | X | QS |
| 18 | Panera Bread | X | X | X | X |  |  |  | FC |
| 19 | IHOP | X | X |  |  | X | X | n/a | FS |
| 20 | Dairy Queen | X | X |  | X |  |  |  | QS |
| 21 | Red Lobster | X | X |  | X | X | X | X | FS |
| 22 | Outback Steakhouse | X |  |  |  |  | X |  | FS |
| 23 | Denny's | X | X |  | X | X | X | X | FS |
| 24 | Papa John's | X |  |  |  |  |  |  | QS |
| 25 | Cracker Barrel Old Country Store |  | n/a | n/a | n/a |  | n/a | n/a | FS |
| 26 | Chipotle Mexican Grill | X |  |  |  |  |  | n/a | FC |
| 27 | T.G.I. Friday's | X | X |  |  | X |  | n/a | FS |
| 28 | Buffalo Wild Wings Grill & Bar |  | n/a | n/a | n/a |  | n/a | n/a | FS |
| 29 | Hardee's | X | X | X | X |  | X | X | QS |
| 30 | Golden Corral | X |  | X |  |  |  | n/a | FS |
| 31 | Popeyes Louisiana Kitchen | X | X |  |  | X | X |  | QS |
| 32 | Ruby Tuesday | X | X |  |  | X | X |  | FS |
| 33 | The Cheesecake Factory |  | n/a | n/a | n/a |  | n/a | n/a | FS |
| 34 | Quiznos | X | X |  | X |  | X | X | QS |
| 35 | Panda Express | X | X |  |  | X | X | n/a | FC |
| 36 | Carl's Jr. | X | X | X | X |  | X | X | QS |
| 37 | Texas Roadhouse |  | n/a | n/a | n/a |  | n/a | n/a | FS |
| 38 | Little Caesars | X |  |  |  |  |  | n/a | QS |
| 39 | Whataburger | X |  | X | X |  | X | X | QS |
| 40 | Red Robin Gourmet Burgers | X |  |  | X |  |  |  | FS |
| 41 | Bob Evans | X | X |  |  | X | X |  | FS |
| 42 | P.F. Chang's | X |  |  |  |  |  |  | FS |
| 43 | Longhorn Steakhouse | X | X |  |  |  |  | X | FS |
| 44 | Hooters |  | n/a | n/a | n/a | X | n/a | n/a | FS |
| 45 | Church's Chicken | X | X |  |  |  |  |  | QS |
| 46 | Waffle House |  | n/a | n/a | n/a |  | n/a | n/a | FS |
| 47 | Steak n Shake | X | X |  |  |  |  |  | QS |
| 48 | Zaxby's | X | X | X | X |  |  |  | FC |
| 49 | Perkins Restaurants & Bakery | X |  |  | X | X |  | n/a | FS |
| 50 | Old Country Buffet/HomeTown Buffet | X |  | X |  | X |  | X | FS |
| 51 | Jimmy John's Gormet Sandwich Shop | X |  |  | X |  | X | n/a | QS |
| 52 | Bojangles' Famous Chicken 'N Biscuits |  | n/a | n/a | n/a |  | n/a | n/a | QS |
| 53 | Culver's Frozen Custard | X | X |  | X | X |  | n/a | QS |
| 54 | Long John Silver's | X | X | X |  | X | X | n/a | QS |
| 55 | Papa Murphy's Take N' Bake Pizza | X | X |  |  |  |  |  | QS |
| 56 | Carrabba's Italian Grill |  | n/a | n/a | n/a |  | n/a | n/a | FS |
| 57 | Logan's Roadhouse |  | n/a | n/a | n/a | X | n/a | n/a | FS |
| 58 | Five Guys Burgers and Fries | X | X |  |  |  |  | n/a | FC |
| 59 | California Pizza Kitchen | X | X |  |  |  |  |  | FS |
| 60 | Friendly's | X | X | X |  | X | X | n/a | FS |
| 61 | Del Taco | X |  |  |  |  |  | X | QS |
| 62 | Romano's Macaroni Grill | X | X |  |  | X |  |  | FS |
| 63 | Baskin-Robbins | X | X | X |  | X | X | n/a | QS |
| 64 | El Pollo Loco | X | X | X | X | X | X |  | FC |
| 65 | O'Charley's | X |  |  |  |  |  | n/a | FS |
| 66 | CiCi's Pizza | X |  | X |  |  |  | n/a | QS |
| 67 | White Castle | X | X | X |  |  |  | X | QS |
| 68 | BJ's Restaurant & Brewhouse |  | n/a | n/a | n/a |  | n/a | n/a | FS |
| 69 | Boston Market | X |  |  |  | X |  | n/a | FC |
| 70 | Jason's Deli | X | X |  |  |  |  | n/a | FC |
| 71 | Sbarro |  | n/a | n/a | n/a |  | n/a | n/a | QS |
| 72 | Ryan's Grill, Buffet & Bakery | X |  | X |  | X |  | X | FS |
| 73 | Ruth's Chris Steak House |  | n/a | n/a | n/a |  | n/a | n/a | FS |
| 74 | In-N-Out Burger | X | X |  |  |  |  |  | QS |
| 75 | Famous Daves |  | n/a | n/a | n/a |  | n/a | n/a | FS |
| 76 | Krispy Kreme | X | X |  |  |  |  |  | QS |
| 77 | Qdoba Mexican Grill | X |  | X | X | X | X | n/a | FC |
| 78 | Jamba Juice | X |  | X |  |  | X | n/a | QS |
| 79 | Tim Hortons | X | X |  | X | X | X | X | QS |
| 80 | Captain D's Seafood Kitchen | X | X | X | X |  |  | n/a | QS |
| 81 | Chuck E. Cheese's | X | X |  |  |  |  |  | FS |
| 82 | Bonefish Girll |  | n/a | n/a | n/a | X | n/a | n/a | FS |
| 83 | Einsten Bros. Bagels | X | X | X | X | X | X | n/a | FC |
| 84 | On the Border Mexican Grill & Cantina | X | X |  |  |  |  |  | FS |
| 85 | Mimi's Café | X |  |  |  | X |  | X | FS |
| 86 | Cold Stone Creamery | X | X |  |  | X |  | X | QS |
| 87 | Krystal Company | X | X |  |  |  |  |  | QS |
| 88 | Moe's Southwest Grill | X |  |  | X |  | X |  | FC |
| 89 | Maggiano's Little Italy |  | n/a | n/a | n/a |  | n/a | n/a | FS |
| 90 | Shoney's |  | n/a | n/a | n/a |  | n/a | n/a | FS |
| 91 | Round Table Pizza | X | X |  |  |  |  | n/a | QS |
| 92 | Checkers Drive-In Restaurants | X | X |  |  |  |  | X | QS |
| 93 | McAlister's Deli | X |  | X | X | X | X |  | FC |
| 94 | Auntie Anne's | X | X | X |  |  |  | X | QS |
| 95 | Uno Chicago Pizza | X |  | X |  |  | X |  | FS |
| 96 | Wingstop |  | n/a | n/a | n/a |  | n/a | n/a | FC |
| 97 | Godfather's Pizza | X | X |  |  |  |  | n/a | QS |
| 98 | McCormick & Schmick's |  | n/a | n/a | n/a | X | n/a | n/a | FS |
| 99 | Carino's Italian | X | X |  |  |  |  | n/a | FS |
| 100 | Sizzler | X | X |  |  |  | X | n/a | QS |

X = feature available; Blank = feature not available

1 n/a = information not assessed since calorie information was not available

2 Healthy eating section was assessed on all websites, regardless of whether calorie information was available

3 n/a = information not assessed since calorie information was not available OR mobile-formatted website was not available

4 QS = quick service; FC = fast casual, FS = full service (casual dining and fine dining)
